# Secreted IgM deficiency leads to increased BCR signaling that results in abnormal splenic B cell development

**DOI:** 10.1038/s41598-017-03688-8

**Published:** 2017-06-14

**Authors:** Dimitrios Tsiantoulas, Mate Kiss, Barbara Bartolini-Gritti, Andreas Bergthaler, Ziad Mallat, Hassan Jumaa, Christoph J. Binder

**Affiliations:** 1 CeMM Research Center for Molecular Medicine of the Austrian Academy of Sciences, Vienna, 1090 Austria; 2 Dept. of Laboratory Medicine, Medical University of Vienna, Vienna, 1090 Austria; 3 Division of Cardiovascular Medicine, University of Cambridge, Cambridge, CB2 OSZ UK; 4 Institute of Immunology, University Hospital Ulm, Ulm, 89081 Germany

## Abstract

Mice lacking secreted IgM (*sIgM* ^−/−^) antibodies display abnormal splenic B cell development, which results in increased marginal zone and decreased follicular B cell numbers. However, the mechanism by which sIgM exhibit this effect is unknown. Here, we demonstrate that B cells in *sIgM* ^−/−^ mice display increased B cell receptor (BCR) signaling as judged by increased levels of phosphorylated Bruton’s tyrosine kinase (pBtk), phosphorylated Spleen tyrosine kinase (pSyk), and nuclear receptor Nur77. Low dosage treatment with the pBtk inhibitor Ibrutinib reversed the altered B cell development in the spleen of *sIgM* ^−/−^ mice, suggesting that sIgM regulate splenic B cell differentiation by decreasing BCR signaling. Mechanistically, we show that B cells, which express BCRs specific to hen egg lysozyme (HEL) display diminished responsiveness to HEL stimulation in presence of soluble anti-HEL IgM antibodies. Our data identify sIgM as negative regulators of BCR signaling and suggest that they can act as decoy receptors for self-antigens that are recognized by membrane bound BCRs.

## Introduction

Secreted IgM (sIgM) antibodies are produced very early in life. A significant part of total IgM are natural IgM, which are produced by innate B-1 B cells in absence of cognate T cell help. SIgM has a pentameric structure, which allows for strong interactions with its antigens^1, 2^. Besides the well documented protective role of sIgM against invading microbial pathogens^1, 2^, these antibodies exhibit also a crucial regulatory role in B cell homeostasis under unchallenged conditions. For instance, marginal zone (MZ) B cells are increased in mice lacking sIgM antibodies (*sIgM* ^−/−^), whereas follicular (FO) B cells are decreased^3, 4^. However, the mechanisms by which sIgM control splenic B cell differentiation are unknown.

FO and MZ B cells (both termed B-2 cells) arise in the spleen. They display major differences with respect to their surface receptors, activation properties, circulating capacity and anatomical localization in the spleen. For example, in contrast to MZ, FO B cells, express on their surface the low affinity receptor for IgE (CD23), have circulating capacity and thus are the major source of recirculating mature B cells^5^. MZ B cells resign in the marginal zone, express higher levels of the complement receptor 2 (CD21) than FO B cells and respond rapidly to blood-borne antigens^6^.

Both MZ and FO B cells emerge upon maturation of transitional B cells, which have successfully escaped from the bone marrow selection^5^. The major driver of the developmental fate of transitional B cells towards MZ and FO B cells is most likely the self-antigen induced B cell receptor (BCR) signaling strength. BCR is present on the cell surface in two isotypes with identical specificity, the IgM and IgD^7^. However, while IgM can be secreted, IgD is only found in membrane-bound form. It still remains unclear what the effect of BCR signaling strength is in mature B cell development. Some studies suggested that strong BCR signaling promotes FO, whereas weak BCR signaling favors MZ B cell development^5^. For example, mice lacking Bruton’s tyrosine kinase, which is a crucial mediator of BCR signaling, show reduced total numbers of MZ and FO B cells. However, the MZ population seemed to be less affected than the FO population, which led to the conclusion that strong BCR signaling might promote FO and restrict MZ B cell development^8^. On the other hand, it has been shown using a monoclonal BCR tg mouse specific for the self-antigen Thy-1, that in presence of the self-antigen, and thus presumably stronger BCR signaling, MZ B cell is favored over FO B cell development^9^. Notably, we and others have shown that sIgM bind to cellular debris and apoptotic cells^10, 11^, which are prototypic sources of self-antigens. Thus, sIgM could influence BCR signaling strength by modulating the interaction of BCRs with circulating self-antigens.

We here investigated the role of sIgM in BCR signaling and how this impacts splenic B-2 cell maturation. We demonstrate that in absence of sIgM, splenic B-2 cells display increased BCR signaling, which is responsible for the abnormal B cell development. Moreover, we show that antigen specific sIgM limit the exposure of BCRs to self-antigens resulting in reduced BCR activation. Our data indicate that sIgM facilitate proper B cell development by acting as negative regulators of BCR signaling. Mechanistically, sIgM have the capacity to limit the binding of self-antigens to membrane bound BCRs by acting as decoy receptors and thereby modulate BCR signaling.

## Results

### *sIgM*^−/−^ mice display abnormal splenic B-2 cell development

**Figure 1:**
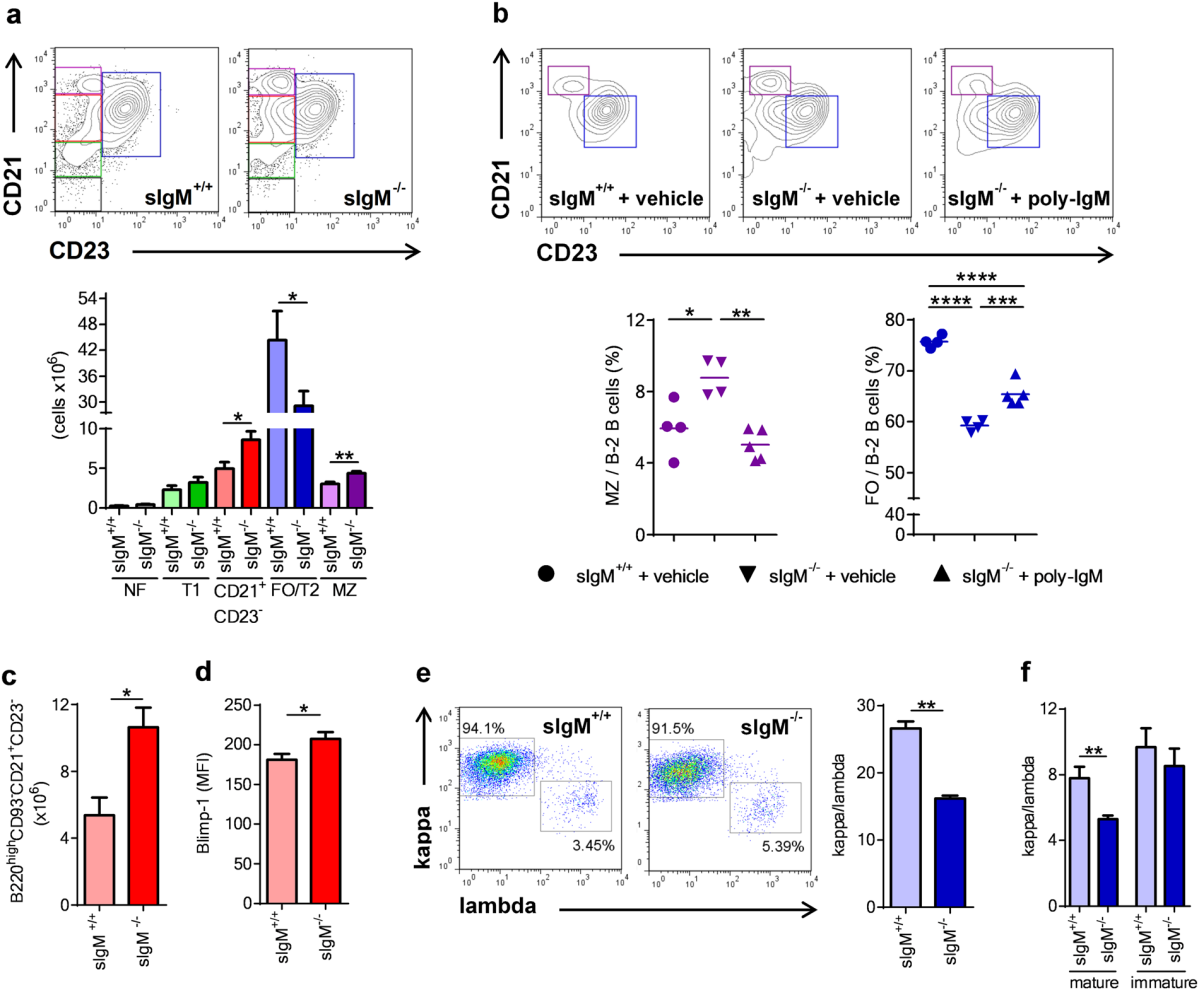
sIgM deficiency results in altered splenic B cell development. (**a**) Representative flow cytometry plots and bar graphs show absolute numbers of FO/T2 (blue), MZ (purple), CD21^+^ CD23^−^ B cells (red), T1 (green) and NF (grey) cells (defined in Fig. S2) of *sIgM* ^+/+^ (light colored bars) and *sIgM* ^−/−^ (dark colored bars) mice. (**b**) Representative flow cytometry plots and dot plots show the frequencies of MZ (purple) and FO/T2 (blue) B cells of *sIgM* ^+/+^ (●) and *sIgM* ^−/−^ (▼) mice treated with vehicle and *sIgM* ^−/−^ (▲) treated with polyclonal IgM (n = 4–5 mice per group). (**c**) Absolute numbers of splenic B220^high^CD93^−^CD21^+^ CD23^−^ B cells and (**d**) Blimp-1 mean fluorescence intensity (MFI) in CD21^+^ CD23^−^ B cells of *sIgM* ^+/+^ (light red bar) and *sIgM* ^−/−^ (dark red bar) mice analyzed by flow cytometry. (**e**,**f**) Representative flow cytometry plots show the percentages of either kappa or lambda light chain positive and bars represent the mean kappa/lambda light chain ratio of (**e**) B220^+^CD43^−^ splenic cells and (**f**) mature (B220^high^ CD43^−^) and immature (B220^low^ CD43^−^) bone marrow cells within cells that express BCR. (**a**,**c**–**f**) Data shown are from one representative experiment of at least two to three independent experiments with 5–6 mice per group. All results show mean ± SEM. *P < 0.05, **P < 0.01, ***P < 0.001, ****P < 0.0001 (Mann-Whitney or unpaired t test or One-Way Anova test followed by Tukey’s test).

In agreement with previous reports^3, 4^ we found increased numbers of marginal zone (MZ) and CD21^+^ CD23^−^ B cells, while CD23 expressing transitional stage 2 and follicular (FO/T2) B cells were strongly decreased in *sIgM* ^−/−^ mice (Fig. 1a and Fig. S1). Newly formed (NF) and transitional stage B cells were not different between *sIgM* ^−/−^ and *sIgM* ^*+/+*^ mice (Fig. 1a and Fig. S1). Moreover, short-term infusion of wild-type polyclonal IgM into *sIgM* ^−/−^ mice could partially reverse the increased MZ and decreased FO B cells (Fig. 1b), which suggests that sIgM influence B cell development during differentiation in the spleen. In line with this, CD21^+^ CD23^−^ B cells remained increased in *sIgM* ^−/−^ mice even after excluding transitional B220^+^CD93^+^ B cells from the analysis (Fig. 1c). This suggests that there is an accumulation of mature B cells at this stage, suggesting an impaired ability to differentiate towards FO B cells. Consistent with this, we found that CD21^+^ CD23^−^ B cells in *sIgM* ^−/−^ mice display increased Blimp-1 levels (Fig. 1d), which is consistent with our previous work showing that increased Blimp-1 levels result in suppression of CD23 expression in B cells^12^. Interestingly, we also found that splenic B cells of *sIgM* ^−/−^ mice display a decreased kappa to lambda light chain ratio compared to *sIgM* ^+/+^ mice (Fig. 1e). However, while similar data were obtained in mature circulating B cells in the bone marrow (Fig. 1f), immature bone marrow B cells exhibit an equivalent kappa to lambda light chain ratio in *sIgM* ^−/−^ and *sIgM* ^+/+^ mice (Fig. 1f). These data suggest that the abnormal splenic B cell maturation is not due to an altered BCR repertoire occurring upon BCR editing in the bone marrow level. Because we found no differences in total numbers of splenic B220^+^IgM^+^ (*sIgM* ^+/+^, 58 ± 9 × 10^6^; *sIgM* ^−/−^, 54 ± 6 × 10^6^), CD19^+^ (*sIgM* ^+/+^, 59 ± 9 × 10^6^; *sIgM* ^−/−^, 62 ± 7 × 10^6^), and CD19^+^7-AAD^+^ dying (*sIgM* ^+/+^, 3 ± 0.6 × 10^6^; *sIgM* ^−/−^, 3 ± 0.5 × 10^6^) B cells, we hypothesized that the abnormal B cell development may be due to altered BCR signaling strength, which is the key driver of splenic B cell development towards MZ or FO B cells^5, 13^.

### Secreted IgM deficiency results in enhanced BCR signaling

**Figure 2:**
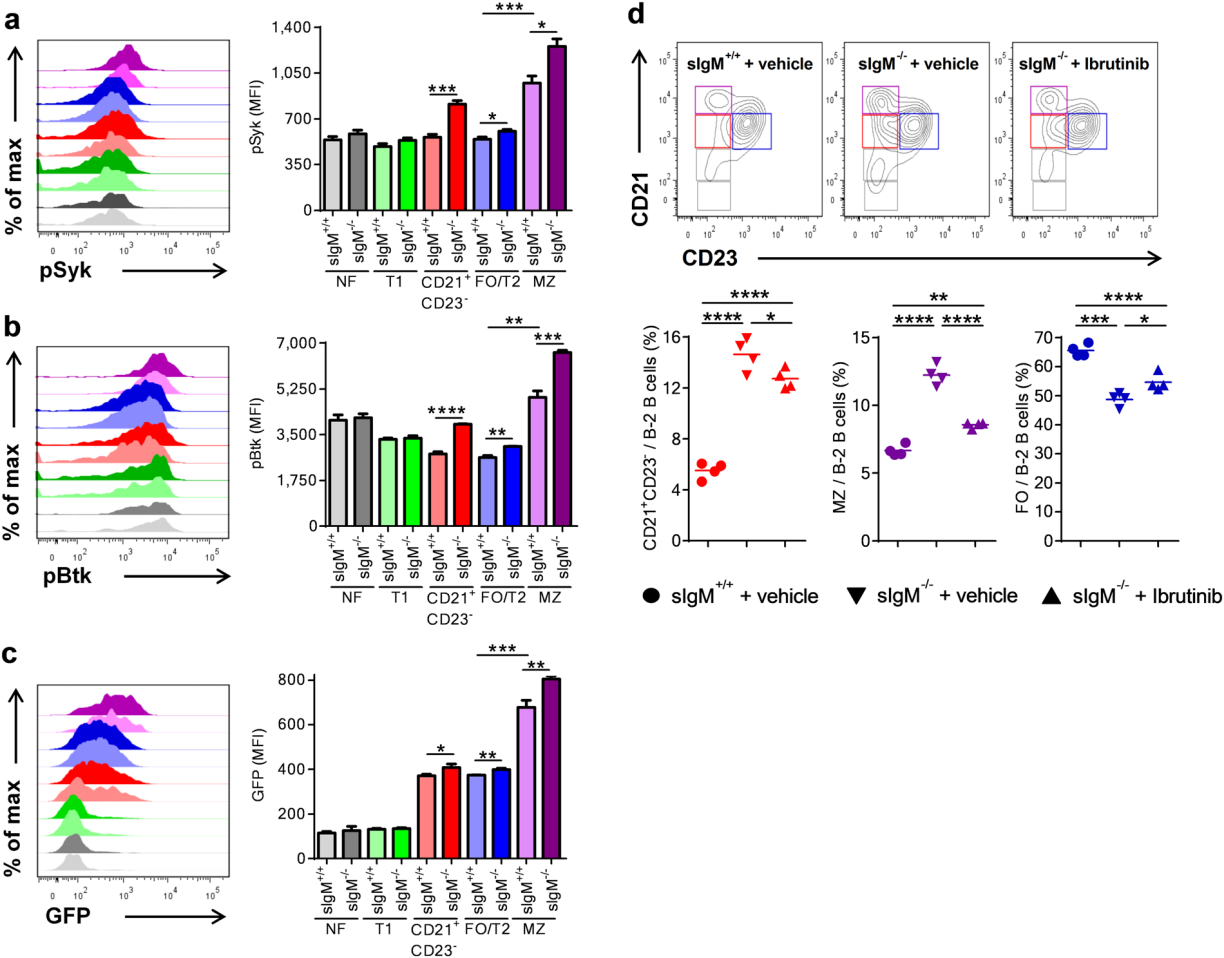
sIgM deficiency results in increased B cell receptor signaling *in vivo*. Representative flow cytometry plots and bar graphs show (**a**–**c**) the mean fluorescence intensity (MFI) for pSyk, pBtk levels and Nurr77-GFP expression in FO/T2 (blue), MZ (purple), CD21^+^ CD23^−^ (red), T1 (green) and NF (grey) B cells (as defined in Fig. S2) of *sIgM* ^+/+^ or *Nur77-GFP*/*sIgM* ^+/+^ (light colored bar) and *sIgM* ^−/−^ or *Nur77-GFP*/*sIgM* ^−/−^ (dark colored bar) mice. Data shown are from one representative experiment (**a**,**b**) or pooled (**c**) from two/three independent experiments. (**d**) Representative flow cytometry plots and dot plots show the frequencies of CD21^+^ CD23^−^ (red), FO (blue) and MZ (purple) B cells within B-2 cells of *sIgM* ^+/+^ (●) and *sIgM* ^−/−^ (▼) mice treated with vehicle, and *sIgM* ^−/−^ (▲) mice treated with the Btk inhibitor Ibrutinib. All results show mean ± SEM, n = 4–6 mice per group. *P < 0.05, **P < 0.01, ***P < 0.001, ****P < 0.0001 (unpaired or paired t test or One-Way Anova test followed by Tukey’s test).

To investigate possible differences in BCR signaling, we quantified the levels of phosphorylated spleen tyrosine kinase (pSyk) and Bruton’s tyrosine kinase (pBtk) in splenic B cell subsets of *sIgM* ^+/+^ and *sIgM* ^−/−^ mice by flow cytometry. These kinases have been shown to be critically involved in antigen mediated BCR signaling and B cell maturation towards FO and MZ B cells^8, 14, 15^. Both MZ and FO/T2 exhibited higher levels of both pSyk and pBtk in *sIgM* ^−/−^ mice. Similar results were obtained for CD21^+^ CD23^−^ splenic B cells (Fig. 2a,b). Notably, NF and T1 B cells, which rely mainly on “tonic” (antigen independent) BCR signaling for their survival^5^, displayed similar levels of pSyk and pBtk in *sIgM* ^+/+^ and *sIgM* ^−/−^ mice (Fig. 2a,b). To confirm our results, we quantified the expression of the nuclear receptor Nur77 as a marker of BCR signaling intensity^16^ in splenic B cell subsets of *Nur77-GFP*/*sIgM* ^−/−^ and *Nur77-GFP*/*sIgM* ^+/+^ reporter mice. Consistent with the increased levels of pBtk and pSyk, we found higher Nur77 expression in MZ, FO/T2 and CD21^+^ CD23^−^ B cells of *sIgM* ^−/−^ mice, whereas NF and T1 B cells showed no difference (Fig. 2c). These data show that mature B-2 cells display increased BCR signaling in *sIgM* ^−/−^ mice.

To investigate further whether enhanced BCR signaling is the cause of abnormal B cell maturation in absence of sIgM, we treated *sIgM* ^−/−^ mice with a low dose of the Btk inhibitor Ibrutinib for two weeks. Vehicle treated *sIgM* ^−/−^ and *sIgM* ^+/+^ mice served as controls. Ibrutinib treatment^17^ did not alter body weight (*sIgM* ^−/−^ + vehicle, 25.7 ± 1.6 g; *sIgM* ^−/−^ + Ibrutinib 26.1 ± 0.6 g) or total B-2 cell numbers (*sIgM* ^−/−^ + vehicle, 56 ± 2 × 10^6^; *sIgM* ^−/−^ + Ibrutinib 50 ± 2 × 10^6^), but rescued the abnormal B-2 cell differentiation kinetics in *sIgM* ^−/−^ mice by decreasing the frequencies of CD21^+^ CD23^−^ B cells and MZ B cells, and concomitantly increasing the frequency of FO B cells (Fig. 2d). Notably, Ibrutinib did not affect NF (*sIgM* ^−/−^ + vehicle, 2.3 ± 0.3%; *sIgM* ^−/−^ + Ibrutinib, 2.3 ± 0.4% out of B220^+^ CD43^−^ B cells) and T1 (*sIgM* ^−/−^ + vehicle, 6.6 ± 0.8%; *sIgM* ^−/−^ + Ibrutinib, 7.3 ± 0.9% out of B220^+^ CD43^−^ B cells) B cells. These data indicate that sIgM prevent excessive BCR signaling in mature B-2 cells and thereby facilitate their proper differentiation towards MZ and FO B cells.

**Figure 3:**
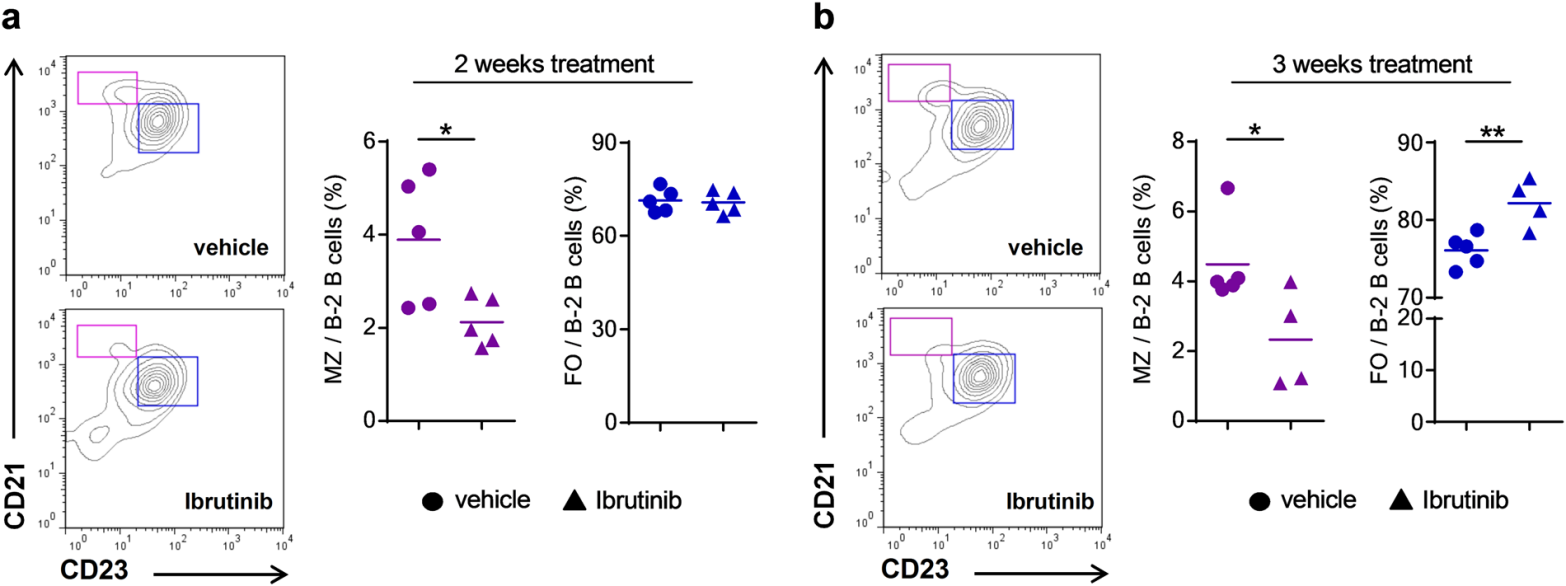
Ibrutinib treatment limits MZ and favors FO B cell development in sIgM^+/+^ mice *in vivo*. Representative flow cytometry plots and dot plots represent the frequencies of MZ (purple) and FO (blue) B cells within B-2 cells of *sIgM* ^+/+^ mice treated with vehicle (●) or the Btk inhibitor Ibrutinib (▲) for (**a**) 2 weeks or (**b**) 3 weeks. All results show mean ± SEM, n = 4–5 mice per group. *P < 0.05, **P < 0.01 (unpaired t test).

Importantly, our data also suggest that strong BCR signaling promotes MZ over FO B cell development. Indeed, our analyses of BCR signaling demonstrate that MZ B cells (light purple bar) display substantially higher pSyk and pBtk levels than FO/T2 B cells (light blue bar) in *sIgM* ^+/+^ mice (Fig. 2a,b). Similarly, Nur77 expression was higher in MZ B cells (light purple bar) compared to FO/T2 B cells (light blue bar) in *Nur77-GFP*/*sIgM* ^+/+^ reporter mice (Fig. 2c). To investigate this further, we treated *sIgM* ^+/+^ mice with Ibrutinib for 2 and 3 weeks and assessed the distribution of MZ and FO B cell in the spleen. Ibrutinib treatment in s*IgM* ^+/+^ mice, did not affect their body weight (2-week treatment; s*IgM* ^+/+^ + vehicle, 21.2 ± 0.7 g; s*IgM* ^+/+^ + Ibrutinib 19.5 ± 0.7 g and 3-week treatment s*IgM* ^+/+^ + vehicle, 20.8 ± 0.37 g; s*IgM* ^+/+^ + Ibrutinib 21 ± 0.5 g) and total B-2 cell numbers (2-week treatment; s*IgM* ^+/+^ + vehicle, 68 ± 10 × 10^6^; s*IgM* ^+/+^ + Ibrutinib 68 ± 8 × 10^6^ and 3-week treatment; s*IgM* ^+/+^ + vehicle, 108 ± 3 × 10^6^; s*IgM* ^+/+^ + Ibrutinib 98 ± 12 × 10^6^). However, compared to vehicle treated controls Ibrutinib treatment of s*IgM* ^+/+^ mice lead to an altered B-2 cell differentiation profile, characterized by a robust reduction of MZ B cells already after 2-weeks treatment and a proportional increase of FO B cells that became evident after 3-weeks treatment (Fig. 3a,b). These data suggest that lowering BCR signaling limits MZ and promotes FO B cell development also in s*IgM* ^+/+^ mice.

### Antigen-specific secreted IgM dampen self-antigen induced BCR signaling

**Figure 4:**
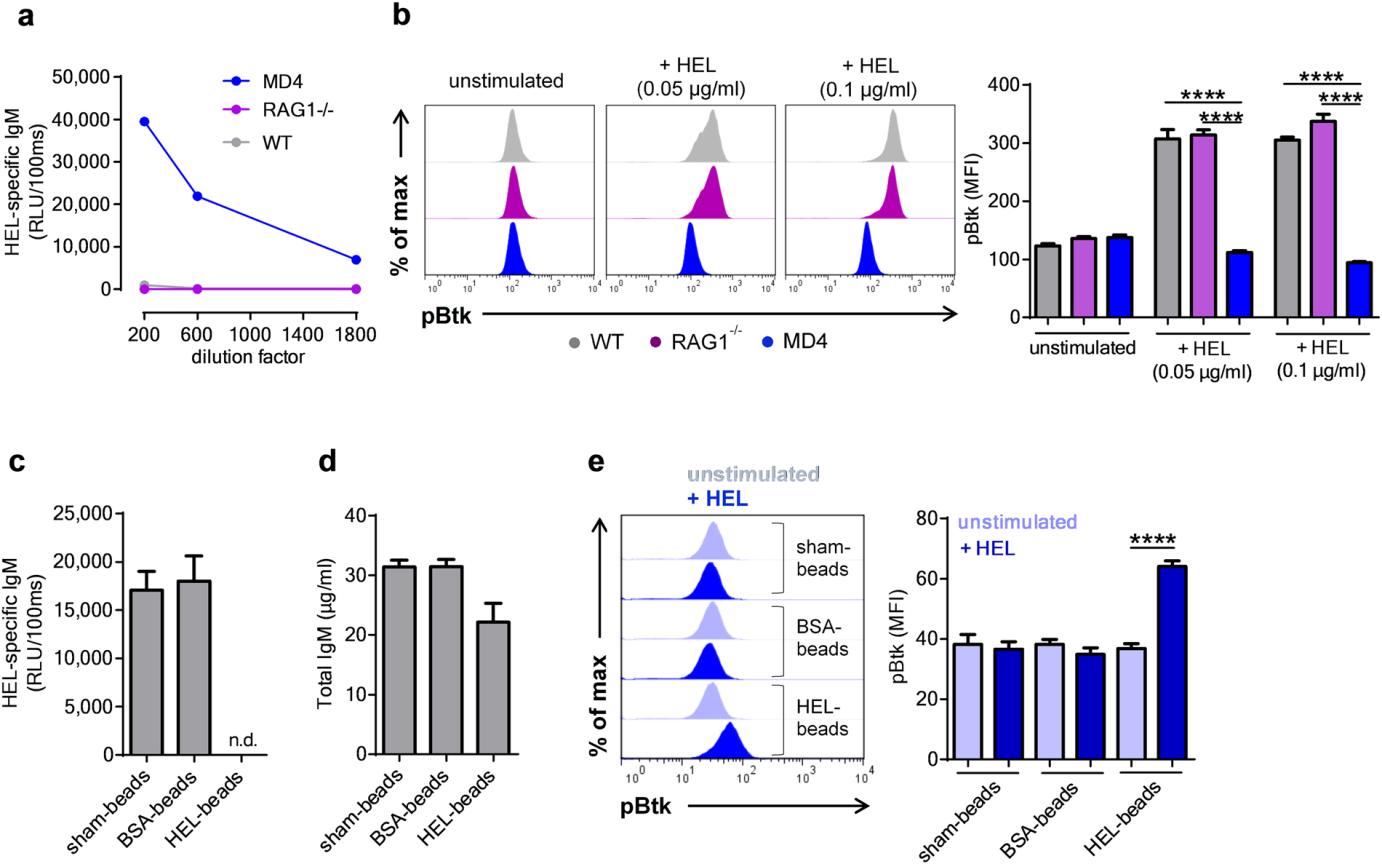
Antigen-specific secreted IgM limit self-antigen induced B cell receptor signaling. (**a**) Quantification of hen egg-white lyoszyme (HEL) specific IgM in the plasma of MD4, RAG1^−/−^ and wild-type (WT) mice by ELISA. (**b**) Representative flow cytometry plots and bar graphs represent the mean fluorescence intensity (MFI) for pBtk in purified B-2 (B220^+^ CD43^−^) cells from MD4^+/−^ mice stimulated with HEL for 3 minutes in presence of either WT (grey), RAG1^−/−^ (purple) or MD4 plasma (blue). ELISA quantification of (**c**) HEL-specific and (**d**) total IgM in MD4 plasma treated with either unconjugated or BSA- or HEL-conjugated microspheres. (**e**) Representative flow cytometry plots and bar graphs show the MFI for pBtk in purified B-2 cells from MD4^+/−^ mice stimulated with HEL for 3 minutes in the presence of plasma from MD4 mice that has been treated with either unconjugated or BSA- or HEL-conjugated microspheres. All results show mean ± SEM, ****P < 0.0001 (unpaired t test or One-Way Anova followed by Tukey’s test). n.d.: not detechtable.

Because sIgM recognize different types of self-antigens such as cellular debris and apoptotic cells^10, 11^, we investigated if sIgM antibodies modulate BCR signaling in a self-antigen mediated manner. For this purpose, we employed the model of MD4 transgenic mice that express BCRs directed against hen egg lysozyme (HEL). MD4 B cells produce HEL-specific IgM antibodies (Fig. 4a), while they lack the ability to class switch to other immunoglobulin isotypes^18^. Stimulation of purified MD4 B-2 cells with HEL in the presence of plasma from either WT (grey bars) or RAG1^−/−^ (purple bars) mice resulted in a robust BCR activation as measured by increased levels of pBtk (Fig. 4b). In contrast, HEL stimulation in presence of plasma from MD4 mice (blue bars) failed to trigger BCR signaling (Fig. 4b). These data suggest that HEL-specific IgM antibodies present in MD4 plasma prevent self-antigen induced BCR activation. To investigate this further, we depleted HEL-specific IgM antibodies from MD4 plasma using microspheres coated with HEL protein. Treatment with HEL-coated microspheres resulted in a complete depletion of HEL-specific IgM, but only a moderate reduction of total IgM, whereas sham-treated or BSA-coated microspheres had no effect (Fig. 4c,d). Notably, MD4 plasma depleted of HEL-specific IgM failed to inhibit HEL-dependent BCR stimulation in purified MD4 B-2 cells, while this was not the case for MD4 plasma treated with either sham-treated or BSA-coated microspheres (Fig. 4e). Thus, sIgM have the capacity to limit self-antigen induced BCR signaling in an antigen specific manner.

## Discussion

The mechanism by which sIgM exhibits its regulatory role in mature B-2 cell development is not known. We demonstrate here that sIgM facilitate proper BCR signaling and thereby physiological differentiation of splenic mature B-2 cells. Mechanistically, we demonstrate that antigen-specific sIgM have the capacity to reduce BCR signaling by limiting the exposure of self-antigens to BCR.

BCR signaling strength is a major determinant of the developmental fate of mature splenic B cells^5^. Data on how BCR signaling strength influences MZ and FO B cell development are contradictory and numerous studies suggest a direct association between BCR signaling strength and FO B cell differentiation. In contrast, our findings indicate that strong BCR signaling favors MZ and restricts FO B cell development. We show that MZ B cells display increased pBtk, pSyk and Nur77 expression than FO B cells *in vivo*. Furthermore, low-dose Ibrutinib treatment, which blocks Btk and lowers BCR signaling, promoted FO and restricted MZ B cell formation in s*IgM* ^+/+^ mice. In addition, we demonstrate that increased BCR signaling in *sIgM* ^−/−^ mice results in an expansion of MZ and a reduction of FO B cells, which was reversed upon Ibrutinib treatment. Thus, our data document that MZ and FO B cell differentiation is promoted by a constitutively strong and weak BCR signaling, respectively.

Although, weak BCR signaling has been proposed to be the cause of impaired FO B cell generation in mice lacking Btk or PLC-γ2^13, 19^, it is important to note that in these mice MZ B cell numbers seem to be less^13^ or even not affected^19^. This suggests that reduced FO B cell development in this setting might be a result of impaired survival rather than disturbed B cell differentiation *per se*, likely due to complete absence of Btk. Along this line, B cell specific deletion of the Lyn kinase, which would increase BCR signaling and according to the current view would promote FO B cell formation, results in strong reduction of both FO and MZ B cells^20^, which further supports the hypothesis that complete lack of such kinases impacts predominately cell survival and thus does not allow safe conclusions on their role with respect to B cell differentiation. On the other hand, we provide evidence that reducing the phosphorylation of Btk *in vivo* with a low dosage of Ibrutinib shifts the differentiation towards FO at the expense of MZ B cells.

CD21^+^ CD23^−^ B-2 cells, which were increased in *sIgM* ^−/−^ mice may function as a reservoir for the development of FO and MZ B cells depending on the BCR signaling strength. In line with this, the increased numbers of CD21^+^ CD23^−^ B-2 cells in *sIgM* ^−/−^ mice may provide an explanation as to why Ibrutinib treatment altered the frequency of FO B cells in these mice already after 2 weeks compared to s*IgM* ^+/+^ mice. Interestingly, we found that in s*IgM* ^+/+^ mice these cells display basal BCR signaling similar to FO B cells, suggesting a higher propensity to differentiate towards FO B cells. In *sIgM* ^−/−^ mice, CD21^+^ CD23^−^ B-2 cell display higher BCR signaling compared to FO B cells, which may not be permissive for differentiation towards FO B cells resulting in their accumulation at the CD21^+^ CD23^−^ stage or their preferential differentiation towards MZ B cells. This hypothesis is supported by our findings that CD21^+^ CD23^−^ B cells of *sIgM* ^−/−^ mice display increased levels Blimp-1, which has been shown to be expressed to a higher extent in MZ compared to FO B cells^21^ and to suppress the expression of the FO B cell marker CD23^12^.

We demonstrate that BCR signaling *in vivo* is significantly increased in the absence of sIgM suggesting that sIgM are negative regulators of BCR signaling. Although a previous study proposed decreased basal BCR signaling in isolated CD43^−^ splenic B cells of *sIgM* ^−/−^ mice based on reduced pErk levels^22^, it has to be kept in mind that phosphorylation of Erk in B cells can be induced via multiple pathways independent of BCR signaling^23^. Furthermore, we did not find any differences with respect to T1 B cells between of *sIgM* ^−/−^ and s*IgM* ^+/+^ mice, which is in contrast to a previous study that reported reduced T1 B cell numbers in *sIgM* ^−/−^ mice^4^. This may be a consequence of different flow cytometry gating strategies by Ngyuen *et al*. who included a large population of CD23^+^ cells in the analysis of T1 B cells^4^. Notably, T1 B cells do not express CD23 and thus inclusion of this population would result in a reduction in T1 B cells concomitant with a reduction in CD23^+^ B cells as it is the case in *sIgM* ^−/−^ mice. Interestingly, splenic B cells of *sIgM* ^−/−^ mice display decreased and increased levels of IgD and IgM BCRs respectively^24, 25^. However, enhanced or diminished signaling via the IgM or IgD-BCR respectively, cannot explain the differences in B cell development seen in *sIgM* ^−/−^ mice, as IgD deficient mice, which still express IgM BCRs and sIgM display normal splenic B cell development^26^. Furthermore, it has been reported that B cells isolated from *sIgM* ^−/−^ mice display similar responsiveness to stimulation with anti-IgM Fab fragments compared to *sIgM* ^+/+^ B cells[22], which further supports that altered surface IgM/IgD expression does not seem to be responsible for the altered BCR signaling.

An alternative possibility by which sIgM influence the B-2 cell development is via the IgM receptor (FcµR). However, several independently generated IgM receptor deficient mouse models have yielded inconsistent results with respect to B-2 cell development that do not resemble the phenotype of *sIgM* ^−/−^ mice^27^. For example, it has been shown in two independent studies that mice deficient in FcµR develop reduced MZ^27^, whereas in a recent study B cell specific deletion of FcµR lead to increased FO B cells^28^. These are in contrast with the increased MZ and decreased FO B cells seen in *sIgM* ^−/−^ mice. Furthermore, we show mechanistically that HEL-specific sIgM are able to prevent HEL-induced BCR activation of MD4 B cells, whereas non HEL-specific IgM, which can still bind to the FcµR, failed to do so. In agreement with this, FcµR receptor, which is also expressed in the trans-Golgi network, affects tonic B cell receptor signaling by regulating the amount of surface bound IgM^28^. Taken together, we conclude that the sIgM-FcµR signaling axis is not responsible for the disturbed splenic B cell development and altered BCR signaling in *sIgM* ^−/−^ mice. In fact, our data suggest that naive B-2 cells secrete IgM that limit their exposure to the antigens they recognize in an antigen-specific manner. Although, identities of the self-antigens that influence B cell development still remain elusive, our study is in agreement with recent reports suggesting that the majority of mature naïve B cells express autoreactive BCRs^16^. With respect to this, it would be particularly interesting to investigate the effect on BCR signaling and B cell developmental fate in *sIgM* ^−/−^ mice that were reconstituted with a poly-IgM preparation that is depleted of IgM with specificity for certain self-antigens. Such assay would provide interesting insights into the regulatory effect of antigen specific soluble IgM in preventing autoreactive B cell activation.

A large amount of total plasma IgM is B-1 cell derived, which have been suggested to display a different and limited repertoire compared to B-2 cells^2^. On the other hand, it should be noted that B-2 cell derived IgM may also contribute significantly to the diversity of the polyclonal IgM pool. Nevertheless, the limited repertoire of B-1 derived IgM may contain specificities that are capable of limiting self-antigen mediated BCR signaling in B-2 cells. It is also conceivable that sIgM could present self-antigens to B cells by recognizing an epitope that is different from the one the BCR recognizes and thereby increase BCR signaling. However, this would not be in line with our findings showing increased BCR signaling in absence of sIgM. In addition, it has been shown that absence of secreted IgM leads to increased autoantibody production^4, 29^, which would be consistent with B cell overactivation in response to an increased free self-antigen pool. Further investigations are required to demonstrate the antigen sequestering properties of secreted IgM *in vivo*.

In conclusion, our data indicate that sIgM are important regulators of BCR signaling strength and suggest that they exhibit these properties in part by functioning as decoy receptors of membrane bound BCRs in an antigen-specific manner. These findings may have broader pathophysiological implication considering that we recently demonstrated that impaired B cell functions in *sIgM* ^−/−^ mice lead to robustly increased plasma IgE, which accelerate atherosclerosis in these mice^30^.

## Materials and Methods

### Mice and treatments

*sIgM* ^−/−^ (on 129 background) and RAG1^−/−^ (on C57BL/6 background) mice were originally bought from The Jackson Laboratories (USA). *SIgM* ^−/−^ mice were backcrossed onto C57BL/6 background for at least 10 generations. MD4 ^Ikzf1(fl/+)^ mice were a kind gift from Dr. Busslinger (IMP, Vienna, Austria). *Apoe* ^−/−^/*Nur77-GFP* reporter mice^31^ (on C57BL/6 background) were kindly provided by Dr. Norbert Gerdes (LMU, Munich, Germany). *Nur77-GFP*/*sIgM* ^−/−^ mice were generated by intercrossing *Apoe* ^−/−^/*Nur77-GFP* and *sIgM* ^−/−^ mice. Experiments were performed only with *Nur77-GFP*/*sIgM* ^−/−^ and *Nur77-GFP*/*sIgM* ^+/+^ mice that were either *Apoe* ^+/+^ or *Apoe* ^+/−^. All mice were bred in our in-house breeding facility. All experiments were performed with age and sex matched mice. Experiments were done with mice between 11–20 weeks of age. All experimental studies were approved by the Animal Ethics Committee of the Medical University of Vienna (Austria) BMWF-66.009/0157-II/3b/2013 and BMWFW-66.009/0030-WF/V/3b/2016. All experiments were performed according to the guidelines for Good Scientific Practice of the Medical University of Vienna (Austria).

### Flow cytometry

Bone marrow cells were isolated from the tibia and the femur bones on cell strainers with 100 µm diameter (BD Biosciences), and erythrocytes were lysed upon incubation with erythrocyte lysis buffer (MORPHISTO). Isolated spleens were mechanically dissociated in single cell suspensions using cell strainers with 100 µm diameter (BD Biosciences), and erythrocytes were lysed as above. Cells were added in a 96 well V-bottom plate (Thermo Scientific) and incubated for 20 min at 4 °C, with 2.5 µg/ml of a blocking anti-CD16/32 antibody (clone 93; eBiosciences) diluted in DPBS (Sigma) containing 10% FBS (FACS buffer). After two washing steps with FACS buffer (393 g for 3 minutes at 4 °C), cells were stained with different combinations of the following antibodies: anti-B220 PercP-Cy5.5 (clone RA3-6B2; eBiosciences), anti-CD23 FITC, anti-CD23 eFluor450 (clone B3B4; eBiosciences), anti-CD43 PE (clone S7; BD Biosciences), anti-IgM APC, anti-IgM FITC (clone II/41; eBiosciences), anti-CD21 biotinylated (clone 7E9; Biolegend), anti-CD93 PE (clone AA4.1; eBiosciences), anti-CD19 PE (clone 1D3; BD Biosciences), anti-kappa FITC (clone 187.1; BD Biosciences), anti-lambda biotinylated (clone RML-42; Biolegend), streptavidin APC or streptavidin eFluor 450 (eBiosciences).

To determine the amount of Blimp-1 and of phosphorylated kinases pBtk and pSyk, cells were fixed and permeabilized with fixation and permeabilization solution (Miltenyi or eBiosciences) for 30 minutes at 4 °C and then stained intracellularly in permeabilization buffer (Milteny or eBiosciences) with the following antibodies: anti-Blimp-1 Alexa Fluor 647 (clone 5E7; BD Biosciences), pBTK/ITK (Y551/Y511) APC (clone M4G3LN; eBiosciences) and pSYK (Y348) APC (clone moch1ct, eBiosciences). Finally, to identify dead cells staining with 7-AAD viability solution (eBiosciences) was performed where indicated. Data were acquired on a FACS Calibur (BD Biosciences) or LSR Fortessa (BD Biosciences) and were analyzed using Flow Jo software 7.6 (Treestar).

### Total and hen egg-white lysozyme specific IgM ELISA

Total and HEL specific IgM in plasma were measured by ELISA. Briefly, 96-well white round-bottomed MicroFluor microtiter plates (Thermo Lab systems) plates were coated with either 5 µg/ml of an anti-mouse IgM (Sigma; M8644) or with 1 µg/ml of HEL (Sigma) in DPBS overnight and then washed 3 times with PBS/EDTA and blocked with Tris-buffered saline containing 1% BSA (TBS/BSA) for 1 h at room temperature. After washing the plates as before, diluted murine plasma was added in TBS/BSA to the wells and incubated for 1 hour at room temperature. Plates were washed and bound total or HEL-specific IgM were detected with an anti-mouse IgM antibody conjugated to alkaline phosphatase (Sigma; A9688). Wells were washed again as before and rinsed once with distilled water, and 25 µl of a 30% LumiPhos Plus solution in dH_2_0 (Lumigen Inc) was added. After 75 min the light emission was measured with a Synergy 2 luminometer (BIO-TEK) and expressed as RLU per 100ms.

### Polyclonal IgM treatment

Female *sIgM* ^−/−^ mice (n = 5) were injected intraperitoneally six times, every two days for two weeks with 200 µg/mouse of polyclonal IgM (Rockland) diluted in 100 µl DPBS (Sigma) and compared to *sIgM* ^−/−^ (n = 4) and *sIgM* ^+/+^ (n = 4) mice that were injected with DPBS only. At the end of the treatment mice were sacrificed and flow cytometric analysis of splenic B cell subsets was performed.

### Ibrutinib treatment

*sIgM* ^−/−^ mice were treated with the Btk inhibitor Ibrutinib (PCI-32765; 25 mg/kg/day/mouse; n = 4) diluted in drinking water containing 5% D-Mannitol (Sigma) and 0.5% gelatin (Sigma) or vehicle only (n = 4) for 2 weeks by oral gavage. Control *sIgM* ^+/+^ mice (n = 4) were treated with the vehicle only. In two independent experiments *sIgM* ^+/+^ mice (n = 5) were treated for 2 or 3 weeks with the vehicle or Ibrutinib (n = 4–5) as above. All mice were fasted for 2 hours before every administration. At the end of the treatment mice were sacrificed and flow cytometric analysis of splenic B cell subsets was performed.

### MD4 B cell stimulation with hen egg-white lysozyme (HEL)

Untouched B-2 cells from MD4^+/−^ mice were purified with the B cell isolation kit (Miltenyi), and purified MD4 B cells (3 × 10^5^/well) were stimulated in triplicates with different concentrations of HEL (Sigma), in the presence of either wild-type, RAG1^−/−^ or MD4 plasma diluted 1:10 in RPMI supplemented with 10% FBS and 1% penicillin/streptomycin for 3 minutes at 37 °C. In some experiments, MD4 plasma was treated with either unconjugated, BSA or HEL-conjugated polystyrene microspheres (Polysciences) to absorb HEL-specific antibodies and used at a dilution of 1:30. Protein conjugation on the surface of microspheres was achieved using the glutaraldehyde method according to the manufacturer’s instructions. At the end of the stimulation, cells were immediately fixed, permeabilized and stained for B220 and pBtk as described above.

### Statistical analyses

Statistical analyses were performed using Graph Pad Prism 5 for Windows (Graph Pad Software). Experimental groups were compared using two tailed Student’s unpaired or paired t test or Mann-Whitney U test as appropriate. To analyze multiple group data One-Way ANOVA test followed by Tukey’s test was performed. Data are presented as mean ± SEM. A P value of <0.05 was considered significant.

## Electronic supplementary material

Supplementary Material
