# Supplementary material for: Secreted IgM deficiency leads to increased BCR signaling that results in abnormal splenic B cell development
Source: Sci Rep. 2017 Jun 14;7:3540. doi: 10.1038/s41598-017-03688-8 (PMC5471202; doi:10.1038/s41598-017-03688-8)
Supplement: Supplementary file 1 — Supplementary Material [file 41598_2017_3688_MOESM1_ESM.pdf]

Secreted IgM deficiency leads to increased BCR signaling that results in abnormal splenic B cell development

Dimitrios Tsiantoulas[1,2,*], Mate Kiss[1,2], Barbara Bartolini-Gritti[1,2], Andreas Bergthaler[1], Ziad Mallat[3], Hassan Jumaa[4], Christoph J. Binder[1,2,*]

Supplementary Materials

Figure S1

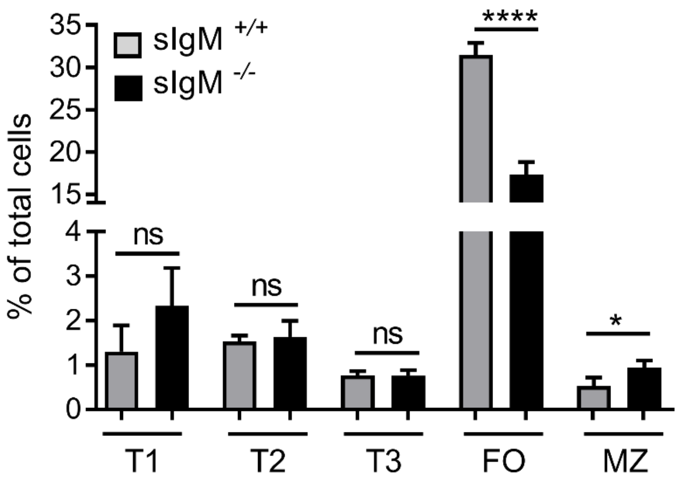

Figure S1. Secreted IgM deficiency results in altered splenic mature B cell development. Bar graph shows T1 ($B220^+IgM^+CD93^+CD21^{low}CD23^-$), T2 ($B220^+IgM^{high}CD93^+CD21^+CD23^+$), T3 ($B220^+IgM^+CD93^+CD21^+CD23^+$), FO ($B220^+IgM^+CD93^-CD21^+CD23^+$) and MZ ($B220^+IgM^+CD93^-CD21^{high}CD23^-$) B cells of *sIgM*$^{+/+}$ (grey bars) and *sIgM*$^{-/-}$ (black bars) mice (n=4 mice per group). Data are shown as mean ± SEM and are representative of two independent experiments. (unpaired t test; * P<0.05, **** P<0.0001).

Figures S2

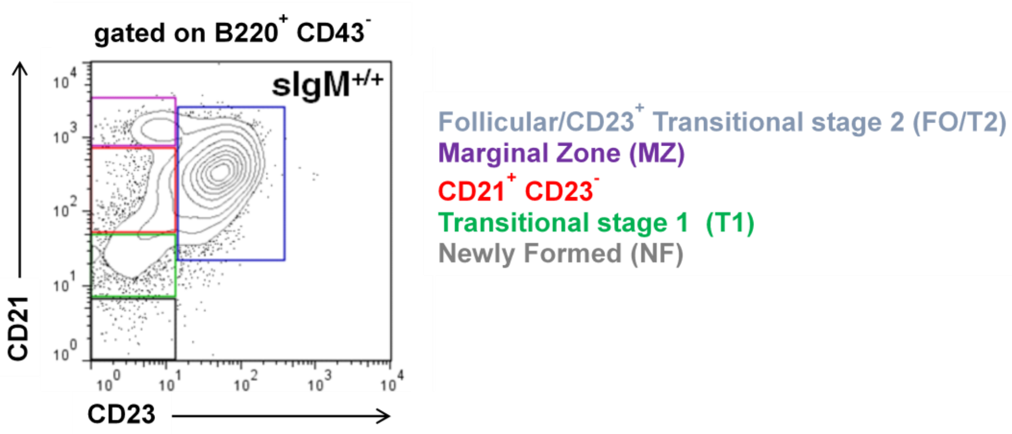

Figures S2. Flow cytometry gating strategy of splenic B cells.

Splenic B cell subsets: follicular and CD23^{+} transitional stage 2 B cells (FO/T2; blue) are defined as B220^{+}CD21^{+}CD23^{+}CD43^{-}, marginal zone (MZ; purple) B cells as B220^{+}CD21^{high}CD23^{-}CD43^{-}, CD21^{+}CD23^{-} B cells as B220^{+}CD21^{+}CD23^{-} CD43^{-} (red), transitional stage 1 (T1; green) B cells as B220^{+}CD21^{low}CD23^{-}CD43^{-} and newly formed (NF; grey) B cells as B220^{+}CD21^{-}CD23^{-}CD43^{-}. To assess only FO B cells, CD21^{high}CD23^{+} B cells were excluded from the gate (not shown).
